# Conformity analysis to demonstrate reproducibility of target volumes for Margin-Intense Stereotactic Radiotherapy for borderline-resectable pancreatic cancer

**DOI:** 10.1016/j.radonc.2016.08.001

**Published:** 2016-10

**Authors:** Daniel L.P. Holyoake, Maxwell Robinson, Derek Grose, David McIntosh, David Sebag-Montefiore, Ganesh Radhakrishna, Neel Patel, Mike Partridge, Somnath Mukherjee, Maria A. Hawkins

**Affiliations:** a CRUK/MRC Oxford Institute for Radiation Oncology, University of Oxford, UK; b The Beatson West of Scotland Cancer Centre, Glasgow, UK; c University of Leeds, CRUK Leeds Centre, UK; d Leeds Cancer Centre, St James’s University Hospital, Leeds, UK; e The Churchill Hospital, Oxford, UK

**Keywords:** Pancreatic cancer, Borderline-resectable, SBRT, Target volume definition, Radiation therapy quality assurance

## Abstract

**Background and purpose:**

Margin-directed neoadjuvant radiotherapy for borderline-resectable pancreatic cancer (BRPC) aims to facilitate clear surgical margins. A systematic method was developed for definition of a boost target volume prior to a formal phase-I study.

**Material and methods:**

Reference structures were defined by two oncologists and one radiologist, target structures were submitted by eight oncologist investigators and compared using conformity indices. Resultant risk of duodenal bleed (NTCP) was modelled.

**Results:**

For GTV, reference volume was 2.1 cm^3^ and investigator mean was 6.03 cm^3^ (95% CI 3.92–8.13 cm^3^), for boost volume 1.1 cm^3^ and 1.25 cm^3^ (1.02–1.48 cm^3^). Mean Dice conformity coefficient for GTV was 0.47 (0.38–0.56), and for boost volume was significantly higher at 0.61 (0.52–0.70, *p* = 0.01). Discordance index (DI) for GTV was 0.65 (0.56–0.75) and for boost volume was significantly lower at 0.39 (0.28–0.49, *p* = 0.001). NTCP using reference contours was 2.95%, with mean for investigator contour plans 3.93% (3.63–4.22%). Correlations were seen between NTCP and GTV volume (*p* = 0.02) and NTCP and DI (correlation coefficient 0.83 (0.29–0.97), *p* = 0.01).

**Conclusions:**

Better conformity with reference was shown for boost volume compared with GTV. Investigator GTV volumes were larger than reference, had higher DI scores and modelled toxicity risk. A consistent method of target structure definition for margin-directed pancreatic radiotherapy is demonstrated.

For patients with a diagnosis of pancreatic cancer surgical resection is the only chance of achieving long-term disease control, yet less than 20% will have resectable disease at diagnosis [1]. Even for resected patients receiving adjuvant chemotherapy, median survival is only around 24 months [2] suggesting there is significant room for improvement by use of optimised multi-modality therapy including neoadjuvant radiotherapy.

In the largest multi‐national adjuvant trial in pancreatic cancer (involving over 1000 patients) positive surgical margins were seen in >35% and were associated with poor outcome [2]. Resection margin status is a strong independent prognostic indicator [3] and survival for patients with positive margins may be little better than for those with unresectable disease [4], though reported rates of microscopic margin involvement depend greatly on histopathological techniques [5].

Borderline-resectable pancreatic cancer (BRPC) is a radiological definition to classify tumours that can be surgically excised but with likely requirement for vascular reconstruction and particularly high risk of positive resection margins [6]. In UK high-volume specialist surgical centres the R1 rate for patients undergoing pancreatectomy with vein resection was 62.9% (144/230) and almost half of these were due to disease at the infiltrated mesenteric vessels [7]. The definition of BPRC is controversial with National Comprehensive Cancer Network (NCCN) criteria being the most widely accepted.

The management of BPRC is also controversial as there are few prospective trials and several therapeutic algorithms have been explored: chemotherapy, radiation and chemoradiation. Current radiation technology permits exquisite dose painting and the possibility to deliver different radiation doses to adjacent areas in the target. Delivering a higher dose to the vessels could be therefore achieved with the aim to sterilize the margin in the area at highest risk, and Stereotactic Body Radiation Therapy (SBRT) offers the opportunity of delivering an ablative dose of RT with short overall treatment time. Retrospective institutional studies have demonstrated the feasibility of such an approach with standard [8], [9] or hypofractionation [10], [11] but in the case of SBRT a systematic method of defining the margin at risk has not been defined.

SPARC (UKCRN ID: 18496) is a CRUK-funded phase 1 dose-escalation study of pre-operative Margin-Intense Stereotactic Radiotherapy for patients with BRPC using the NCCN criteria [12], approved by a National Health Service Research Ethics Committee. SPARC incorporates a comprehensive radiotherapy quality assurance programme to ensure consistency in target definition and radiotherapy delivery. This includes a radiotherapy manual with atlas, and pre-trial contouring and planning test-cases followed by a workshop, both of which have been shown to reduce variation in target volume definition [13], [14], [15]. The radiotherapy manual specifies that the target structure for the margin-directed boost should be defined following discussion with the radiologist and/or Hepato-Pancreato-Biliary (HPB) surgeon to identify the vascular structures that are responsible for the tumour being classified as borderline resectable according to the NCCN criteria.

We aim to describe a novel method of defining the margin at risk for radiotherapy planning, testing of the applicability of this method, and exploration of the implications for Normal Tissue Complication Probability (NTCP) when SBRT is used.

## Materials and methods

**Fig. 1:**
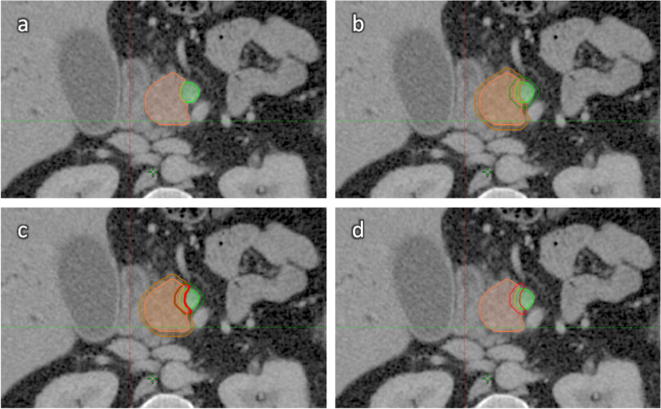
Definition of target volumes for SPARC trial. Orange = GTV, Green = VesselContact, Red = Boost volume.

On an intravenous contrast-enhanced exhale breath-hold CT (CECT) scan of a suitable test-case of BRPC a set of reference structures were defined by a team of two expert clinical oncologists and one radiologist. A contemporaneous ^18^FDG-PET scan was used to help interpret CT appearances but the GTV was contoured to define the extent of gross tumour as evident on the CT scan. The target structure for the margin-directed boost was generated in a stepwise manner (see Fig. 1)**:**

(a) The vessel(s) e.g. superior mesenteric artery, superior mesenteric vein or portal vein should be outlined for their length that they are in contact with the tumour. This structure is denoted VesselContact.
(b) This structure is then expanded circumferentially by 3 mm (i.e. anterior/posterior and laterally but not cranio-caudally). The resulting structure is denoted Vessel + 3 mmC. The GTV is also expanded circumferentially by 3mm to produce GTV + 3mmC.
(c) A Boolean operator is used to define the region that lies in both Vessel + 3mmC AND GTV + 3mmC, and the resultant structure is denoted the Boost Volume.

Eight clinical oncologist investigators specialising in pancreatic cancer were provided with the CT and PET scans, along with radiologist reports, and asked to follow the written instructions for the delineation of the target structures within the radiotherapy guidance for the SPARC trial protocol. Structure sets were imported into the Eclipse (version 13, Varian, Palo Alto, CA) radiotherapy treatment planning system (TPS) and descriptive parameters (volume, centre of mass) and conformity indexes were calculated:

$$\text{Dice coefficient}=\frac{2\times (A\cap B)}{A+B}$$

$$\text{Geographical miss index}=\frac{B-(A\cap B)}{B}$$

$$\text{Discordance index}=1-\left(\frac{\left(A\cap B\right)}{A}\right)$$

where A denotes the investigator structure and B the reference structure, and A∩B denotes intersection of A and B (equivalent to Boolean operator “A AND B”).

The trial protocol mandates the use of motion mitigation techniques if motion is greater than 5 mm (for example abdominal compression), followed by a 4DCT or fiducial markers and tracking. The CECT is used to confirm the exhale position when reviewing the 4DCT and the tumour and at risk volumes are contoured on each phase of the 4DCT to derive the ITV. A 3 mm 3D margin is then added to create the PTV. On line volumetric image verification is mandated. An SBRT plan was produced based on the reference set of target structures, to deliver 35 Gy to the primary tumour PTV and 50 Gy to the margin at risk (the highest dose level). Treatment is delivered in five daily fractions. This plan was then used to reproducibly create plans for each set of investigator target structures, and cumulative dose–volume histogram (DVH) statistics were extracted describing target coverage and Organ at Risk (OAR) exposure. The dose–volume constraints for the duodenum are: *D*_10cc_ < 25 Gy, *D*_9cc_ < 15 Gy, *D*_5cc_ < 25 Gy, *D*_1cc_ < 33 Gy, *D*_max (0.5cc)_ < 35 Gy. NTCP for risk of upper gastrointestinal bleed in the duodenum was modelled using the Lyman-Kutcher Burman model within the Biological Evaluation Module of the TPS, adopting parameters derived by Pan et al. (TD_50_ = 180, *m* = 0.49, *n* = 0.12, *α*/*β* ratio = 3) [16]. Shapiro–Wilk testing did not show evidence of deviation from normality, hence Pearson’s product-moment correlation testing and 2-tailed paired t-tests were performed using Rstudio [17], and a threshold of *p* < 0.05 was defined as significant.

## Results

**Fig. 2:**
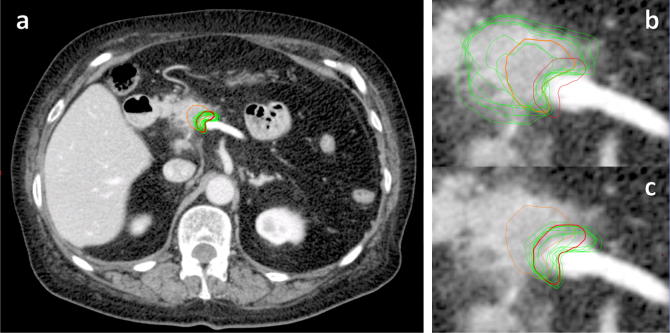
Reference (orange = GTV, red = boost volume) and investigator contours (green) of boost volume (images a and c) and GTV (image b) on contrast-enhanced axial CT of patient with borderline-resectable pancreatic carcinoma.

**Fig. 3:**
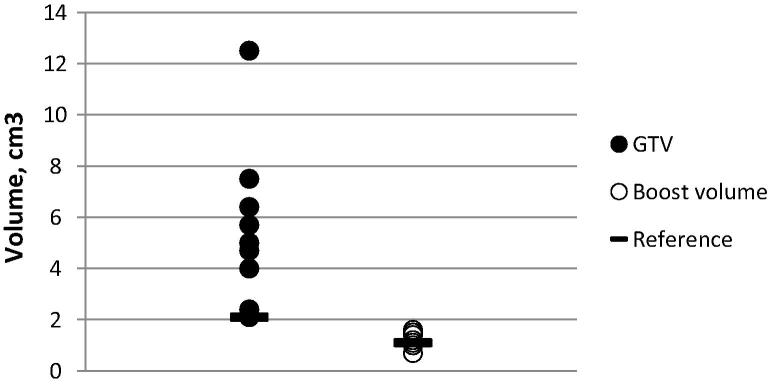
Investigator target structure volumes compared to reference.

**Fig. 4:**
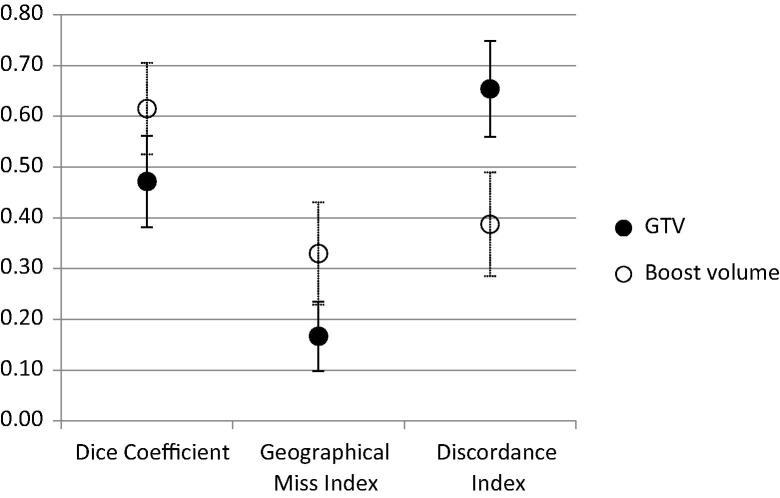
Mean conformity indices for investigator target structures when assessed against reference (error bars show 95% confidence interval).

Reference and mean investigator volumes for GTV were 2.1 cm^3^ and 6.03 cm^3^ (95% CI 3.92–8.13 cm^3^) respectively, and for boost volume were 1.1 cm^3^ and 1.25 cm^3^ (1.02–1.48 cm^3^) (Fig. 2, Fig. 3). Mean Dice conformity coefficients for GTV and boost volume were 0.47 (0.38–0.56) and 0.61 (0.52–0.70), significantly higher for the boost volume (*p* = 0.01); mean discordance indices (DI) were 0.65 (0.56–0.75) and 0.39 (0.28–0.49), significantly lower for the boost volume (*p* = 0.001); mean Geographical Miss Indices (GMI) were 0.17 (0.10–0.23) and 0.33 (0.23–0.43), *p* = 0.005. Correlation coefficient for GTV volume with DI was 0.93 (0.65–0.99, *p* =  < 0.001), and for GTV with GMI was −0.06 (−0.73–0.68, *p* = 0.899) (Fig. 4).

**Fig. 5:**
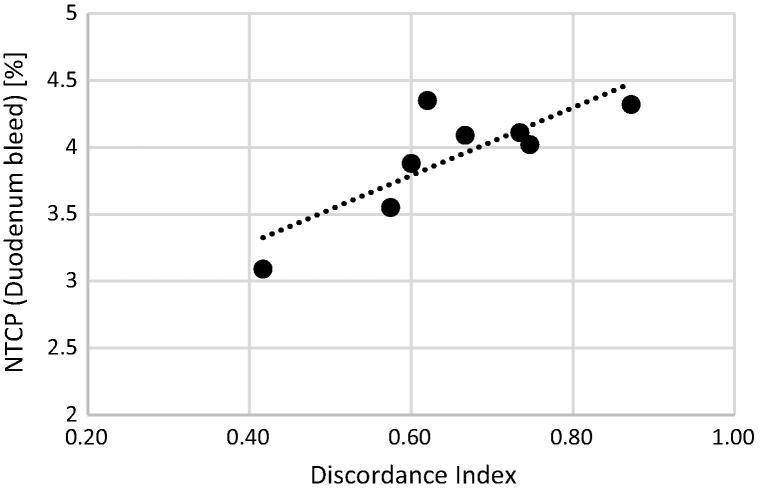
NTCP (risk of duodenal bleed) according to Discordance Index.

Treatment plans based on the investigator contours were able to meet the trial planning constraints for target coverage and OAR avoidance but were associated with an increased risk of NTCP (duodenal bleed): risk for the plan based on reference target structures was 2.95%, while mean risk for plans using investigator structures was 3.93% (3.63–4.22%). Risk of duodenal bleed was correlated with delineated tumour volume (correlation coefficient 0.74, 95% CI 0.15–0.94, *p* = 0.02) and with discordance index (0.83, 0.29–0.97, *p* = 0.01) (Fig. 5).

## Discussion

New techniques in radiotherapy planning, including new target volume concepts, should be subject to rigorous assessment. We believe this is the first analysis of variability in volume delineation for margin-directed SBRT in pancreatic cancer, in which we have demonstrated a method that has led to strong inter-user conformity for definition of the boost volume, in a region of complex anatomy, across multiple centres. No guidelines yet exist for definition of a margin-directed radiotherapy boost in localised pancreatic cancer, but the systematic method for definition of the boost target structure in the SPARC trial protocol has led to high consistency in clinician target volume definition and in preventing inclusion of more tissue within the target volumes than was intended by the trial design.

We have shown that the investigator boost volume structures showed less inter-observer variance in volume compared with the investigator GTV structures, and investigator GTV volumes were also larger than the gold standard, while this was not the case for the boost volume. We have also shown that conformity to reference, as measured by Dice coefficient, was higher for the boost volume than for GTV, and Discordance Index (DI) was higher for GTV, in keeping with ‘over-contouring’. Radiobiological modelling suggests that radiotherapy plans based on the submitted investigator contours would have led to increased risk of clinically important toxicity (duodenal bleed) and that the risk increased not only with size of contoured GTV, as might be expected, but particularly with degree of discordance from reference. Predicted rates of toxicity remain low, and on a similar scale to those seen in a recent phase II study of fractionated pancreatic SBRT with gemcitabine (2% acute and 11% late grade ⩾ 2 upper gastrointestinal toxicity) [18].

The usefulness of the increased conformality achieved by modern radiotherapy depends on accurate target definition by the treating clinician – “There is little point in worrying about how to deliver image-guided modulated arc radiation plans if they are to the wrong target” [19]. Errors in target volume delineation tend to be larger than other geometric errors in radiotherapy planning, and also cause a systematic error, not only for a specific patient but potentially for all patients treated by a given clinician or centre [20]. In theory the GTV should be a factual entity defined by all observers in the same way, but despite this significant inter-observer variability has been recorded in contours of tumours and organs at risk [21] and in our study we have observed a large variation even among a small group of experienced clinicians. In our study we had intentionally used an ‘expert-defined’ reference rather than a mathematically-derived consensus contour, and we feel this has been important in highlighting a systematic pattern of over-contouring, which would have been obscured by the use of a consensus definition.

Measures of volume alone cannot assess spatial discrepancy and thus measures of position should be used, such as through concordance indices which integrate volumetric and positional differences. The Dice coefficient indicates the overall degree of agreement between two contours. Geographical Miss Index (GMI) scores reflect the amount of the reference contour not included in the investigator contour, while (DI) indicates the amount of investigator contour that was not in the reference structure. In our results, DI scores for GTV were higher than those for boost volume, GMI scores were lower for GTV than for boost volume while other conformity indices were similar for the two structures. Generously contoured structures may be less likely to exclude any of the reference structures, and can achieve better GMI scores [22], whereas the DI is useful to highlight incorrectly large target structures, i.e. “too much unnecessary volume” [23]. In our data the GTV volume correlated strongly with DI (*p *< 0.001), as expected, however GTV volume did not show a strong negative correlation with GMI, suggesting that generous contouring did not lead to improved tumour coverage. While the GMI was found to be significantly higher for the boost volume than for the GTV, the values and range (0.33, 95% CI 0.23–0.43), are similar to those reported for the GTV itself in another recent investigation of pancreatic tumour definition (median GMI 0.26, Inter-Quartile Range 0.15–0.40) [24].

Accurate GTV delineation is particularly important in pancreatic radiotherapy due to the proximity of critical structures, however the optimal imaging modalities for visualising pancreatic tumours remain unknown [25]. Pancreatic tumours usually appear hypodense relative to normal pancreas on CECT, but some are isodense and very difficult to define [26] and it is the junction between tumour and normal pancreatic tissue where the most variability has been seen in our results. A diagnostic quality CECT in breath-hold helps target and normal structure visualisation. Multi-modality imaging including FDG-PET and multi-parametric MRI can be used to guide the clinician but the potential effect on outcomes remains uncertain [25]. In addition pathology correlation studies suggest that tumour size is underestimated by some imaging modalities [27], [28]. The value of direct radiologist support in radiotherapy planning is increasingly understood [29] however this does not obviate the requirement for the modern radiation oncologist to be skilled in multi-modality image interpretation and have highly detailed anatomical knowledge in order to safely deliver complex treatments such as described here.

Four prior publications were identified describing the use of margin-directed radiotherapy in pancreatic cancer (Table 1). Chuong et al. were first to publish a report of margin-directed radiotherapy in pancreatic cancer, delivering a simultaneous integrated boost to a PTV “encompassed that portion of tumour adjacent to the vasculature resulting in the borderline designation” [30]. The authors describe that they “tailored the high dose volume on a case per case basis...the tumor vessel interface (1 cm of vessel with 1 cm of tumor) was delineated but also modified based on proximity of critical structures” [10]. A preoperative chemo-radiotherapy regimen adopted by Hirata et al. also included dose escalation, targeted at the roots of the coeliac and superior mesenteric arteries, being at “high risk of perineural invasion but difficult to dissect completely”. The boost target volume was “personalised according to guidance from the surgeons or reduced if necessary to meet duodenal or stomach radiotherapy dose-volume constraints” [9].

In a prospective phase 1 study in patients with Locally Advanced Pancreatic Cancer (LAPC), Passoni and colleagues delivered a simultaneous integrated boost to a target structure encompassing the vessels infiltrating the tumour [8]. In this case a systematic method was used to define the target for the boost: Infiltrating vessels were contoured and expanded by 1 cm; then this structure was trimmed to remain within the primary GTV (in contrast to our approach, in which portions of the boost volume lie outside the primary GTV). In the most recent publication in this field the ‘Vessel Boost’ target was defined as the 5 mm of tumour around the vessel(s) identified that rendered the tumour borderline-resectable, expanded by a further 5 mm for set-up error. This methodology is similar to ours, but in the setting of conventionally fractionated treatment, such a technique has not yet been utilised for SBRT planning [11]. The boost volume we have described does not have any superior or inferior margin added prior to ITV and PTV definition. The rationale is to avoid irradiating part of the vessel that is not in contact with tumour to very high dose. Part of this vessel will be used for vascular reconstruction and the long term effect of SBRT on this tissue is unknown.

In conducting radiotherapy clinical trials, consistency of treatment planning and delivery across patients and recruiting centres is required not only for sound conclusions to be drawn regarding the treatment intervention but also because radiotherapy that is not delivered per-protocol is associated with worse patient outcomes [31]. In a study of chemoradiotherapy for locally-advanced pancreatic cancer, protocol deviations such as unnecessarily large target volumes correlated with risk of significant toxicity [32] while in a large study of adjuvant chemoradiotherapy after resection of pancreatic adenocarcinoma, failure to adhere to the radiotherapy protocol was associated with decreased overall survival [33]. Therefore, it is recommended that radiotherapy trials are supported by a prospective and comprehensive quality assurance programme [20]. We have shown that pancreatic GTV delineation remains difficult, which reinforces the importance of pre-treatment central review of target structures to ensure consistency and quality across recruiting centres.

## Conclusions

We have demonstrated a robust systematic method for delineation of the boost volume for margin-intense pancreatic SBRT, and with the use of a detailed protocol and atlas within a clinical trial setting this has led to less inter-user variability and greater conformity with a reference contour than was observed for definition of the primary tumour GTV. We have also shown variability in GTV definition with implications for modelled toxicity risk, highlighting the difficulties in pancreatic tumour delineation and reinforcing the importance of continuing on-trial RTQA.

## Conflict of interest statement

The authors declare no conflict of interest.

## Figures and Tables

**Table 1:** Published reports of margin-directed radiotherapy in pancreatic cancer.

Reference	Indication	Fractions	Tumour dose	Boost dose	Margin/boost target
Chuong 2013	BRPC or LAPC	5	25–30 Gy	30–40 Gy	Individualised:- tumour-vessel interface
Passoni 2013	LAPC	15	44.25 Gy	48–58 Gy	Systematic:- infiltrating vessels + 1 cm within GTV
Hirata 2015	Resectable PDAC	25	50 Gy	60 Gy	Individualised:- roots of coeliac vessels & SMA
Wang 2015	BRPC or LAPC	28	50.4 Gy	56 Gy	Systematic:- tumour-vessel interface

BRPC = Borderline-Resectable Pancreatic Cancer, LAPC = Locally Advanced Pancreatic Cancer, PDAC = Pancreatic Ductal AdenoCarcinoma, SMA = Superior Mesenteric Artery.
